# Bacterial Purine Nucleoside Phosphorylases from Mesophilic and Thermophilic Sources: Characterization of Their Interaction with Natural Nucleosides and Modified Arabinofuranoside Analogues

**DOI:** 10.3390/biom14091069

**Published:** 2024-08-27

**Authors:** Irina A. Bychek, Anastasia A. Zenchenko, Maria A. Kostromina, Marat M. Khisamov, Pavel N. Solyev, Roman S. Esipov, Sergey N. Mikhailov, Irina V. Varizhuk

**Affiliations:** 1 Engelhardt Institute of Molecular Biology, Russian Academy of Sciences, 119991 Moscow, Russia; 2 Shemyakin-Ovchinnikov Institute of Bioorganic Chemistry, Russian Academy of Sciences, 117997 Moscow, Russia

**Keywords:** biochemistry, purine nucleoside phosphorylases, modified nucleoside analogues, mesophilic enzymes, thermophilic enzymes

## Abstract

The enzymatic synthesis of nucleoside derivatives is an important alternative to multi-step chemical methods traditionally used for this purpose. Despite several undeniable advantages of the enzymatic approach, there are a number of factors limiting its application, such as the limited substrate specificity of enzymes, the need to work at fairly low concentrations, and the physicochemical properties of substrates—for example, low solubility. This research conducted by our group is dedicated to the advantages and limitations of using purine nucleoside phosphorylases (PNPs), the main enzymes for the metabolic reutilization of purines, in the synthesis of modified nucleoside analogues. In our work, the substrate specificity of PNP from various bacterial sources (mesophilic and thermophilic) was studied, and the effect of substrate, increased temperature, and the presence of organic solvents on the conversion rate was investigated.

## 1. Introduction

Purine nucleoside phosphorylases (PNPs) (EC 2.4.2.1) are nucleic acid metabolism enzymes involved in the utilization and reutilization pathways of purine nucleotides. This type of enzyme catalyzes the reversible phosphorolysis of purine nucleosides, both naturally occurring and their modified analogs [1,2,3]. Bacterial PNPs are widely used in the synthesis of nucleoside analogs [4,5], which are employed as antiviral and antitumor agents and have recently attracted increasing attention as antibacterial agents [6,7,8,9,10]. Currently, over 100 drugs are registered with the FDA, of which the active substances are nucleic acid components and their synthetic analogs [9,10].

Nucleoside analogs can be obtained via chemical and enzymatic synthesis methods, or their combination. Enzymatic synthesis, despite all its advantages (regioselectivity, stereospecificity, and environmental friendliness), has a number of drawbacks that complicate its application both in laboratory practice and in industrial production [11]. Among them are the high specificity of enzymes, which markedly narrows the pool of possible substrates, and the low stability of enzymes under harsh conditions, such as high or low pH values, elevated temperatures, and the presence of organic solvents. The use of enzymes from different sources can help to offset these disadvantages, for example, by differing substrate specificity or temperature optima of each individual enzyme.

Therefore, in this work, we studied the substrate specificity of these enzymes in relation to their natural substrates—ribonucleosides and the group of modified nucleosides, arabinonucleosides—on the examples of mesophilic *Escherichia coli* (*Ec*PNP), *Enterobacter* (*Ent*PNP), and thermophilic *Thermus thermophilus* HB27 type I and II (*Tth*PNP I and II) purine nucleoside phosphorylases. Arabinonucleosides were chosen for our study because of their wide application both in antiviral and anticancer therapy (fludarabine, vidarabine, and cytarabine) [10]. Due to the low solubility of many modified nucleosides and bases, in particular arabinonucleosides presented in this work, the stability and preservation of PNP activity under harsh synthesis conditions (elevated temperatures, presence of organic solvents) are of particular importance.

Comparative studies of the activity and basic kinetic parameters of phosphorolysis in the presence of enzymes from various bacterial sources, carried out in the framework of this work, provide the researcher with the necessary information to select the enzyme suitable for their specific conditions of synthesis. The use of PNPs with different characteristics significantly expands the possibilities of enzymatic synthesis through mutual supplementation of the substrate pool and the use of different temperature regimes and organic solvents, providing multilateral optimization of synthesis.

## 2. Materials and Methods

### 2.1. Equipment

Absorption spectra were recorded using a Cary 3500 UV/VIS spectrophotometer (Agilent, Santa Clara, CA, USA). Semi-microcuvettes with a volume of 1400 μL (optical path length 10 mm) made of Suprasil^®^ quartz glass (Hellma, Müllheim, Germany) were used.

HPLC analysis was carried out on a gradient system Akvilon (Podolsk, Russia) in the following configuration: two Stayer pumps (HLP-210) and a Stayer UV detector (UVV-105) on a 4 × 150 mm Phenomenex chromatographic column (5 µm, Luna C18 (2)-100 Å, Part No. 00F-4252-E0, Phenomenex, Torrance, CA, USA).

The pH of the solutions was measured with a microprocessor pH meter pH 211 (Hanna Instruments, Vöhringen, Germany), equipped with a combined electrode HI 1131 and a thermoelectrode HI 7662.

The ^1^H-NMR and ^13^C-NMR spectra were recorded on a Bruker AVANCE II 300 (Billerica, MA, USA) instrument at a temperature of 303 K with an operating frequency of 300.1 MHz for ^1^H and 75.5 MHz for ^13^C. The chemical shifts (δ) are given in parts per million (ppm), measured relative to the residual solvent signal as an internal standard (DMSO-*d*_6_, ^1^H: 2.5 ppm, ^13^C: 39.5 ppm). The spin-spin coupling constants (*J*) are measured in hertz (Hz). When describing ^1^H-NMR spectra, the following abbreviations are used: s = singlet, d = doublet, dd = doublet of doublets, and m = multiplet.

High-resolution mass spectra were recorded on a hybrid quadrupole time-of-flight mass spectrometer, the Bruker Daltonics micrOTOF-Q II (electrospray ionization). Measurements were carried out in positive ion mode under the following conditions: spray capillary voltage 4500 V; mass scanning range m/z 50–3000 Da; external calibration (Electrospray Calibrant Solution, Fluka, Charlotte, NC, USA); nebulizer pressure 0.4 bar; flow rate 3 μL·min^−1^; nebulizer gas—nitrogen (4.0 L·min^−1^); and interface temperature 180 °C. Samples were injected into the spray chamber of the mass spectrometer from an Agilent 1260 high-performance liquid chromatograph equipped with an Agilent Poroshell 120 EC-C18 column (3.0 × 50 mm; 2.7 μm); the flow rate was 0.4 mL·min^−1^; the samples of compounds were loaded from an aqueous acetonitrile solution and eluted in the following gradient of acetonitrile (A) in water: 0–6 min—0%-85% A, 6–7.5 min—85% A, 7.5–8 min—85%-0% A, 8–10 min—0% A.

### 2.2. Chemicals

Acetonitrile (CH_3_CN), HPLC grade; ethanol (C_2_H_5_OH), HPLC grade; potassium dihydroorthophosphate (KH_2_PO_4_); potassium hydroxide (KOH); inosine (Ino); adenosine (Ado); arabinofuranosyladenine (ara-A); hypoxanthine (Hx); tris (hydroxymethyl)-aminomethane (Tris-HCl), Luria-Bertani (LB) medium, ampicillin, isopropyl β-D-thiogalactoside (IPTG), ethylenedinitrilotetraacetic acid (EDTA), benzydamine hydrochloride, phenylmethylsulfonyl fluoride (PMSF), sodium azide (NaN_3_), ammonium sulfate (NH_4_)_2_SO_4_, glycerol were purchased from Sigma–Aldrich (USA) and were of highest available purity. Hydrochloric acid (HCl), reagent grade, was purchased from Reakhim (Moscow, Russia). Dimethyl sulfoxide (DMSO), reagent grade; dimethylformamide (DMF), reagent grade; trifluoroacetic acid (CF_3_COOH), reagent grade; and sodium chloride (NaCl), reagent grade, were purchased from JSC Lenreaktiv (Saint Petersburg, Russia). All samples, buffer solutions, and reaction mixtures were prepared using mQ Millipore-grade water (18.2 MΩ).

### 2.3. Enzymes

Recombinant enzyme preparations of purine nucleoside phosphorylases (PNP, EC 2.4.2.1), xanthine oxidase (XO, EC 1.17.3.2), and adenosine deaminase (ADA, EC 3.5.4.4) were used in the work. PNP from *Enterobacter*, xanthine oxidase from cow’s milk, and adenosine deaminase from calf intestine were purchased from Sigma–Aldrich (St. Louis, MO, USA).

### 2.4. Purification of PNP from Escherichia coli

The producing strain, *E. coli* ER2566/pER-EcPNP, was cultivated at 37 °C in LB medium supplemented with 100 µg/mL ampicillin. Upon reaching the optical density of A_595_ = 0.8, the cell cultures were supplemented with 0.4 mM of IPTG, followed by further cultivation for 4 h at 37 °C. The cell biomass was disrupted in a buffer of 50 mM Tris-HCl pH 8.0, 5 mM EDTA, 1 mM benzydamine hydrochloride, and 1 mM PMSF using an ultrasonicator. A target enzyme was purified from clarified cell lysate by anion exchange chromatography on an XK 16/20 column packed with Q Sepharose FF sorbent (Cytiva, Washington, DC, USA), pre-equilibrated with a buffer of 50 mM Tris-HCl pH 8.0 and 5 mM EDTA. The proteins were eluted with a linear gradient of NaCl (0 to 500 mM). The fractions containing the desired enzyme were pooled, concentrated on the YM-30 membrane (Merck Millipore, Burlington, MA, USA), and applied onto a HiLoad 16/60 Superdex 200 pg column (Cytiva, Washington, DC, USA), pre-equilibrated with buffer 50 mM Tris-HCl pH 8.0, 200 mM NaCl, and 0.04% (*w*/*w*) NaN_3_. Fractions containing the target enzyme with a purity higher than 95% were pooled, concentrated on the YM-30 membrane to the final concentration of 30 mg/mL, and stored at −80 °C [12,13].

### 2.5. Purification of PNP I and II from Thermus thermophilus HB27

The producing strains *E. coli* C3029/pGro7/pER-TthPNP I and C3029/pKJE7/pER-TthPNP II were cultivated at 37 °C in LB medium supplemented with 100 µg/mL ampicillin, 20 µg/mL chloramphenicol, and 0.5 mg/mL L-arabinose. Upon reaching the optical density of A_595_ = 0.8, the cell cultures were supplemented with 0.4 mM of IPTG, followed by further cultivation for 4 h at 37 °C. The cell biomass of both strains was disrupted in a buffer of 50 mM Tris-HCl, pH 8.5, using an ultrasonicator. The clarified cell lysate was heat-treated at 70 °C for 10 min to precipitate contaminating proteins. The *Tth*PNP I purification was performed by metal chelate affinity chromatography on an XK 16/20 column packed with Protino Ni-NTA Resin (Macherey-Nagel, Dueren, Germany) with a step-gradient of imidazole (50 to 250 mM). In the first step, the *Tth*PNP II was salted out of the heat-treated clarified cell lysate with 40% saturation of (NH_4_)_2_SO_4_ and purified by anion exchange chromatography on an XK 16/20 column packed with DEAE Sepharose Fast Flow resin (Cytiva, Washington, DC., USA) pre-equilibrated with buffer 50 mM Tris-HCl, pH 8.5, and 5 mM EDTA in a linear gradient of NaCl (0 to 1 M). In the second step, hydrophobic chromatography was performed on an XK 16/20 column packed with HiTrap Phenyl Sepharose Fast Flow, which was pre-equilibrated with a buffer of 50 mM Tris-HCl, pH 8.5, and 5 mM EDTA in a linear gradient (NH_4_)_2_SO_4_ (0.5 to 0 M). Further purification of both enzymes was carried out according to the same scheme given above for *Ec*PNP. The purification using size-exclusion chromatography was performed in a buffer of 50 mM Tris-HCl, pH 8.0, 5 mM EDTA, 50 mM NaCl, 10% glycerol, and 0.04% (*w*/*w*) NaN_3_. Fractions containing target enzymes with a purity higher than 95% were collected, concentrated to the final concentration of 10.0 mg/mL, and stored at −80 °C [13,14].

The activities of xanthine oxidase and adenosine deaminase were assumed to be equal to those indicated in the supplier’s certificate of analysis. The activity of PNPs from *Enterobacter*, *Escherichia coli*, and *Thermus thermophilus* HB27 was measured in accordance with the methods given below.

### 2.6. Activity Measurements

The activity of all PNPs was determined at pH 7.5 in 50 mM KH_2_PO_4_ buffer at 25 °C for mesophilic and 80 °C for thermophilic enzymes. The concentration of nucleosides is presented in Table 1. All reaction mixtures were prepared in a volume of 1 mL, and reactions were started by the addition of PNPs. The observation wavelength is shown in Table 1. The initial phosphorolysis velocity was calculated by a linear regression of absorbance vs. time. The difference in extinction coefficients between the reaction product and the starting nucleoside used in the experiment was obtained experimentally and shown in Table 1. Initial substrate concentrations were chosen to achieve saturation or were close to the maximum solubility at 25 °C. Experiments were performed at least twice.

When assessing the effect of organic solvents and temperatures on the activity of enzymes in the reaction of phosphorolysis of inosine, in all cases, the measurements were carried out using the direct spectrophotometric method. In the case of the experiment with *Tth*PNP I, carried out spectrophotometrically at 80 °C, it was necessary to make a correction for evaporation-condensation and associated fluctuations in concentrations. For this purpose, solutions identical to the reaction mixtures but without the addition of enzymes were used as a zero control. Experiments were performed at least in triplicate.

To determine the long-term stability of *Ec*PNP, the enzyme solutions in KH_2_PO_4_ (50 mM, pH 7.5) were placed in a thermostat at different temperatures (45, 50, 55, 60, and 65 °C) and kept there constantly for the duration of the experiment. Aliquots of the enzyme solution were taken at appropriate intervals, and the enzyme's activity was measured. The measurements were also carried out by the direct spectrophotometric method (b). Experiments were conducted at least in triplicate for each temperature.

### 2.7. Kinetic Methods

Kinetic curves were plotted based on the initial reaction rates at a fixed phosphate concentration (50 mM, pH 7.5). The calculation was carried out for at least seven concentration points, either until a plateau was reached or until the maximum value was available due to the solubility of the substrate (Table S2). The methods and maximum concentrations used are consistent with the activity determination experiments (Table 1 and Table S2). The experiment for each concentration was carried out at least twice. The temperature used for mesophilic enzymes was 25 °C; for thermophilic ones, it was 80 °C.

Calculation of the kinetic parameters of phosphorolysis in the presence of PNP is usually carried out by fitting them to two models: the interacting site model or the classical Michaelis-Menten model [1,14,15,16,17]. To simplify calculations, the latter was chosen for this comparative study. Curve-fitting was carried out using the GraphPad Prism program v.8.0.2 (GraphPad Software, Boston, MA, USA).

### 2.8. Synthesis of 9-(β-D-Arabinofuranosyl)Hypoxanthine

A sample of 9-(β-D-arabinofuranosyl)adenine (26.7 mg, 0.1 mmol) was dissolved in 20 mL of KH_2_PO_4_ buffer solution (50 mM, pH 7.5). The reaction was initiated by adding 1 IU of ADA. The reaction mixture was kept at 45 °C. The progress of the reaction was monitored using HPLC. After completion of the reaction, the reaction mixture was concentrated in vacuo to a volume of 5 mL. The resulting concentrated solution was applied to a C18 column for separation. Fractions containing the product (determined by a UV detector at λ = 260 nm) were collected separately and evaporated to dryness in vacuo. The reaction yield was 96.5% (25.9 mg). The structure of the resulting compound was confirmed by ^1^H-NMR spectroscopy (Supplementary Materials, Figure S1). ^1^H-NMR (300.1 MHz, DMSO-*d*_6_): δ = 8.14 (s,1H, H8), 8.03 (s, 1H, H2), 6.21 (d, 1H, *J*_1′,2′_ = 4.8 Hz, H1′), 5.65 (s, 1H, 3′-OH), 5.55 (s, 1H, 2′-OH), 5.07 (s, 1H, 5′-OH), 4.16-4.09 (m, 2H, H3′ + H4′), 3.78 (dd, 1H, *J*_2′, 3′_ = 8.9 Hz, H2′), 3.67 (dd, 1H, *J*_5′ a,4′_ = 3.9 Hz, *J*_5′ a, 5′ b_ = −11.7 Hz, H5′ a), 3.63 (dd, 1H, *J*_5′ b,4′_ = 5.0 Hz, H5′ b), corresponds to [18]. ^13^C-NMR (75.5 MHz, DМSO-*d*_6_): δ = 156.68 (C6), 148.11 (C4), 145.67 (C2), 139.50 (C8), 123.44 (C5), 84.20 (C1′), 83.84 (C4′), 75.67 (C3′), 74.71 (C2′), 60.69 (C5′). HRMS: *m/z* [C_10_H_12_N_4_O_5_ + K]^+^ calculated *m/z* 307.0445, found *m/z* 307.0439, [C_10_H_12_N_4_O_5_ + H]^+^ calculated *m/z* 269.0886, found *m/z* 269.0880. UV (H_2_O, pH 7.0): λ_max_, nm (ε, M^−1^ cm^−1^) = 249 (12,000), corresponds to [19].

## 3. Results and Discussion

Enzymatic synthesis is safe, environmentally friendly, and does not require complex purification of products due to the regioselectivity and stereospecificity of enzymatic reactions. However, the enzymatic approach also has several disadvantages. First of all, the exceptional selectivity of enzymes limits the pool of possible substrates. For example, the PNPs exhibit a relatively wide range of specificity towards possible modifications in the nitrogenous base but are more sensitive to modifications in the sugar residue [2,20]. In addition, enzymes, being protein structures, are susceptible to denaturation under harsh conditions. At the same time, many nucleosides and nucleic bases are limitedly soluble in aqueous solutions at moderate pH and temperatures (Table 2, [21]), as are their modified analogs used as medicinal compounds [22].

The disadvantages of enzymatic synthesis can be partially overcome by using enzymes from different sources. The issue of enzyme instability at elevated temperatures, for example, can be solved by using enzymes from thermophilic microorganisms [15,25]. The problem with a limited pool of possible substrates, however, has no easy solution. The ongoing work on the mutation of enzymes to expand their substrate specificity and stability is very encouraging but has not yielded breakthrough results so far [26,27,28,29]. At the same time, the wide distribution of nucleoside phosphorylases among various organisms provides some variation in substrate specificity, which may help to partially solve the problem by mutually supplementing the pool of substrates.

### 3.1. Comparison of Substrate Specificity of Mesophilic and Thermophilic Enzymes towards Natural Substrates and Their Arabino-Derivatives

To carry out a comparative study of enzyme activity towards standard natural substrates and their sugar moiety-modified derivatives, arabinonucleosides were chosen as the latter due to their therapeutic value. The goal of many research groups around the world is to develop an efficient biocatalytic approach to synthesize this particular type of derivative, as they demonstrate both cytotoxic (cytarabine, nelarabine, clofarabine, fludarabine) and antiviral activity (vidarabine) [30]. Reports from different research groups involved in the isolation and study of NPs from mesophilic and thermophilic sources on the efficiency of these enzymes in the synthesis of arabinonucleosides have been rather promising [31,32].

The direct comparison of kinetic parameters of phosphorolysis in the presence of both types of enzymes under standardized conditions was of particular interest. When choosing the conditions of this study, we were guided by the following points: Among the selected enzymes, *Ec*PNP has the broadest substrate specificity; this enzyme uses both 6-oxo- and 6-aminopurines as substrates. *Ent*PNP and *Tth*PNP I have affinity only for 6-oxopurines, and *Tth*PNP II only for 6-aminopurines. Therefore, inosine (Ino) was chosen as the reference substrate for *Ec*PNP, *Tth*PNP I, and *Ent*PNP, and adenosine (Ado) was chosen as the reference substrate for *Tth*PNP II. Arabinohypoxanthine (ara-Hx) and arabinoadenine (ara-A) were chosen as modified substrates. The enzyme activity and kinetic parameters of phosphorolysis were measured at pH 7.5. For mesophilic PNPs (*Ec*PNP, *Ent*PNP), a standard temperature of 25 °C was used. For thermophilic enzymes (*Tth*PNP I and II), a temperature of 80 °C was used. The data obtained are summarized in Table 3.

In the case of phosphorolysis of natural substrates, mesophilic enzymes are characterized by a higher enzyme-to-substrate affinity than thermophilic ones (mean K_M_ values 21–107 vs. 383–894, see Table 3 for SD). This may be explained by the lower flexibility of the active center of thermophilic enzymes due to the need for a more rigid formation of the tertiary structure of the protein capable of maintaining stability at high temperatures [33]. The reaction rate of phosphorolysis in the presence of thermophilic enzymes is higher than in the presence of mesophilic ones (activity and k_cat_ values are in almost all cases higher by an order of magnitude). At the same time, the overall catalysis efficiency (k_cat_/K_M_) is comparable for all studied enzymes.

Calculation and analysis of the kinetic characteristics of the phosphorolysis of arabino derivatives were complicated by the concentration range limited by substrate solubility. The maximum concentrations of substrates in the reaction mixtures were 1.5 mM for ara-A and 4.0 mM for ara-Hx to preserve the possibility of storing stock solutions at room temperature. Kinetic curves at the available concentration ranges do not reach plateau, which significantly reduced the accuracy of the calculation of characteristics (errors were high and exceeded 50%). The curves, however, are instrumental in evaluating the order of magnitude of the K_M_, V_max_, and k_cat_ values and establishing the tendency in the efficiency of catalysis upon the transition from ribo- to arabino derivatives.

Since the calculated values of K_M_, V_max_, and k_cat_ not only are obtained with a significant error but also lie in a concentration range that is not achievable practically even at 80 °C, both the observed constants (marked “obs”, obtained for the highest of the experimentally measured concentrations) and the calculated values (“calc”) are presented in Table 3. Nevertheless, the general tendency for a significant decrease in the efficiency of catalysis upon the transition from ribo- to arabino-derivatives is obvious. The decrease in k_cat_/K_M_ ranged from 3 to 4 orders of magnitude. Activity, in our case equal to V_max obs_, decreases most for *Ent*PNP and *Tth*PNP II. *Ec*PNP loses activity the least among all PNPs studied, being specific for both ara-A and ara-Hx nucleosides.

### 3.2. Effect of Organic Solvents on the Activity of Mesophilic and Thermophilic PNPs

**Figure 1:**
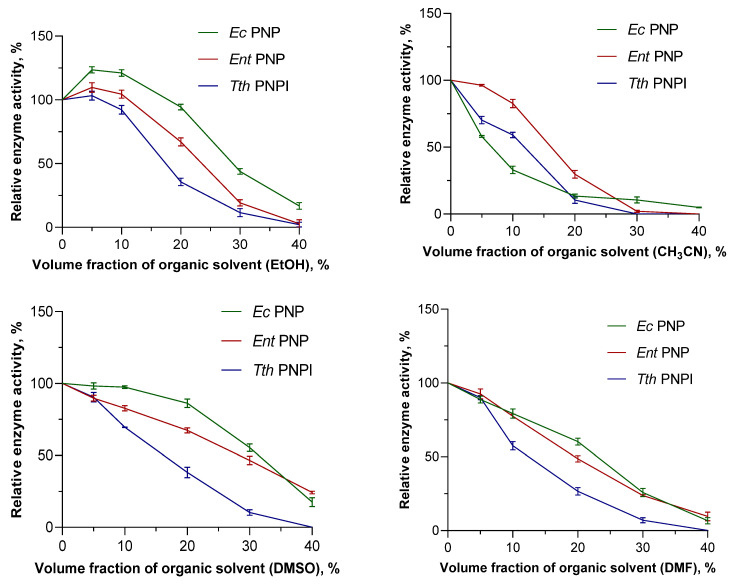
The influence of organic solvents on activity of mesophilic and thermophilic PNPs in the reaction of inosine phosphorolysis (50 mM KH_2_PO_4_, pH 7.5, 25 °C for *Ec*PNP and *Ent*PNP, 80 °C for *Tth*PNP I).

The addition of organic solvents to reaction mixtures is one of the most frequently used methods to solve the problem of low substrate concentration in biocatalysis. However, this approach is limited by the denaturation of proteins in organic media and the loss of their activity. Therefore, a separate step of the comparative work was to investigate the effect of organic solvents on the activity of PNPs. For comparison, we selected *Ec*PNP, *Ent*PNP, *Tth*PNP I, and inosine as their common reference substrates. Acetonitrile (CH_3_CN), dimethylformamide (DMF), ethyl alcohol (EtOH), and dimethyl sulfoxide (DMSO) were considered the most common solvents in laboratory practice and were miscible with water. The phosphorolysis reaction was studied in the solvent volume fraction range of 0 to 40% at 25 °C for *Ec*PNP and *Ent*PNP and at 80 °C for *Tth*PNP I. The effect of organic solvents on catalysis efficiency was evaluated by the change in the activity of PNPs in the presence of different volume fractions of organic solvents. The obtained data are summarized in Table S1 (Supplementary Materials) and Figure 1.

According to the data obtained, *Ec*PNP retains residual activity (5 to 17.6%) at 40% content of all presented solvents. *Ent*PNP completely loses its activity at 40% CH_3_CN content; in the case of other solvents, residual activity is preserved (2.9 to 24.3%). The thermophilic enzyme *Tth*PNP I retained residual activity at 40% organic solvent content in the case of EtOH only. The mesophilic enzymes presented in this work retained 50% activity at 20% solvent content in all cases except CH_3_CN (Table S1).

Among the solvents studied, DMSO and EtOH are the mildest in their effect on enzymes. At small percentages of these solvents (up to 17–24%), EtOH shows better results than DMSO; beyond this level, a milder effect is observed for DMSO. It is also interesting that an increase in the relative activity of each of the presented enzymes was observed for reaction mixtures with EtOH content ranging from 1 to 18%. The maximum of this effect is demonstrated in the case of *Ec*PNP—123% at 5% EtOH.

In general, for the three enzymes studied, mesophilic PNPs are characterized by greater resistance to the addition of organic solvents to the reaction mixture.

### 3.3. Influence of Temperature on the Activity of PNPs

**Figure 2:**
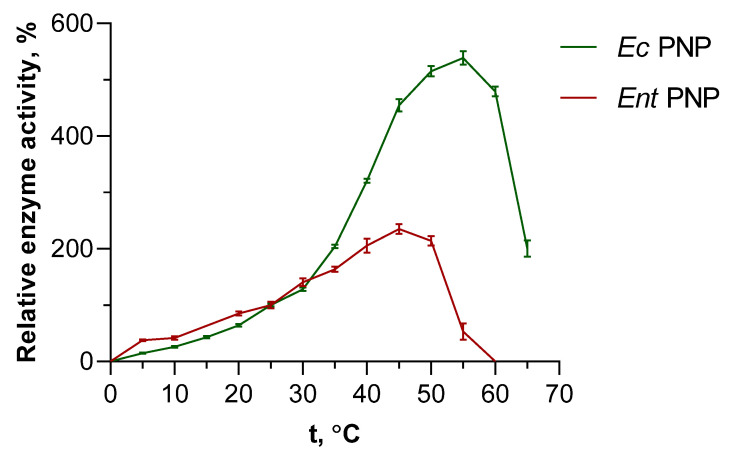
Effect of temperature on *Ec*PNP and *Ent*PNP activity in the reaction of inosine phosphorolysis. Relative enzyme activity is a percentage of the value obtained at 25 °C (50 mM KH_2_PO_4_, pH 7.5).

In addition to accelerating the reaction, increasing the temperature leads to increased solubility of substrates, which may allow optimization of the synthesis. Due to the high interest of researchers in thermophilic enzymes in recent decades, the literature contains relevant and detailed data on their thermal stability. Thus, it is known that thermophilic NPs show an increase in activity from 60 °C up to the boiling point of aqueous solutions (not always, however, maintaining long-term stability at such temperatures) [34,35,36]. *Ec*PNP, as the most frequently used enzyme in enzymatic synthesis, is also well studied and characterized [1,37]. Within the scope of this work, it was of interest to compare *Ec*PNP with another, less studied mesophilic enzyme, *Ent*PNP. Inosine was also chosen as a substrate in the phosphorolysis reaction. The data obtained are demonstrated in Figure 2.

When temperatures rise above 35 °C, a significant increase in *Ec*PNP activity is observed, while the increase in *Ent*PNP activity is more gradual. The maximum activity of *Ec*PNP, estimated to be 540% of the standard activity at 25 °C, is reached at 55 °C. The maximum activity of *Ent*PNP peaked at 45 °C, with 235% values at 25 °C. Beyond 50 °C, a rapid decline of *Ent*PNP activity is observed, reducing to no detectable activity at 60 °C. Activity of *Ec*PNP is decreased slower and smoother with rising temperature, and at 65 °C the activity is still two times higher than at 25 °C.

**Figure 3:**
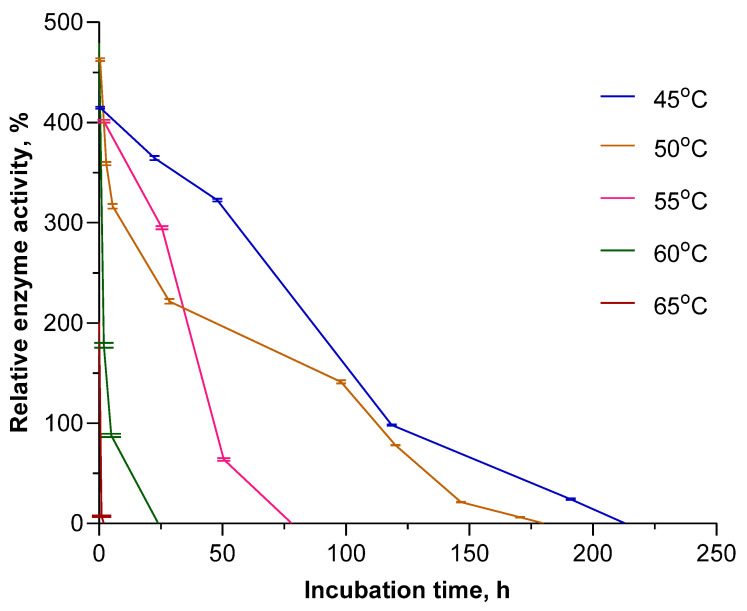
Long-term stability of *Ec*PNP at elevated temperatures. Enzyme activity in the reaction of inosine phosphorolysis (50 mM KH_2_PO_4_, pH 7.5) obtained at 25 °C for freshly prepared enzyme solution taken as 100%.

Having obtained such superior results for *Ec*PNP, we decided to further study the long-term stability of this enzyme in the temperature range of 45–65 °C. Inosine was chosen as the substrate. The enzyme was placed into a thermostat at the appropriate temperature, and its residual activity was measured at certain intervals. The results obtained are shown in Figure 3.

At a temperature of 55 °C, which corresponds to its maximum activity, the enzyme retains activity up to 80 h. However, the increased activity of the enzyme compared to the standard at 25 °C is only observed for the first 40 h. A temperature of 45 °C can be considered optimal, since at this temperature the enzyme not only remains active the longest (up to 213 h) but also, starting from the third hour, has the highest relative activity.

## 4. Conclusions

The study of the phosphorolysis of natural nucleosides and their arabino derivatives in the presence of PNPs from *Escherichia coli*, *Enterobacter*, and *Thermus thermophilus* HB27 allows us to draw some general conclusions. The efficiency of catalysis significantly decreases with the substrate switch from ribo- to arabino-derivatives for all studied enzymes (by 3–4 orders of magnitude). At the same time, when comparing the kinetic characteristics of phosphorolysis reactions, mesophilic enzymes are characterized by a higher enzyme-substrate affinity (K_M_ from 21 ± 5 μM for ribo-derivatives to 2898 ± 831 μM for arabino-) versus thermophilic enzymes (K_M_ from 383 ± 132 μM for ribo-derivatives to ≥21500 μM for arabino-). The turnover numbers of thermophilic enzymes are higher (k_cat_ from 0.42 s^−1^ for arabino-derivatives to 483 s^−1^ for ribo-) than in the case of mesophilic enzymes (k_cat_ from 0.0079 s^−1^ for arabino-derivatives to 138 s^−1^ for ribo-). Mesophilic PNPs demonstrated greater tolerance for the presence of organic solvents in the reaction medium in the example of inosine phosphorolysis. Thus, *Ec*PNP retains 5 to 17.6% residual activity at 40% content of all solvents used; *Ent*PNP maintains 2.9 to 24.3% activity in the presence of all solvents, except for 40% CH_3_CN. *Tth*PNP I shows no activity at 40% solvent content for any of the solvents tested, except 40% EtOH, where residual activity is observed.

In terms of synthetic applications of PNPs, the rich spectrum of available enzymes from various sources provides researchers with a large toolbox with diverse and well-studied characteristics. This allows greater flexibility when choosing a synthesis strategy. For example, thermophilic enzymes are indispensable when it is necessary to work at higher temperatures to increase conversion rates or the solubility of substrates. Mesophilic enzymes, in their turn, are useful when working with labile compounds; they show a higher affinity for substrates while being able to remain functional in a fairly wide range of conditions.

## Figures and Tables

**Table 1:** Spectral data and concentrations of substrates used in activity determination.

Substrate	Concentration	λ_obs_ (nm)	ε or Δε (M^−1^cm^−1^)	Method *	Enzyme
Ino	400 μM	293	12,000	a	*Ec*PNP, *Ent*PNP
	15 mM	278 for b; 260 for c	1100 for b; 7900_Hx_/7100_Ino_ for c	b and c	*Tth*PNP I
Ado	200 μM	274	1020	b	*Ec*PNP
	4 mM	260	13,300_Ade_/14,900_Ado_	c	*Tth*PNP II
Ara-Hx	4 mM	243	9600_Hx_/11,300_araHx_	d	*Ec*PNP, *Ent*PNP, *Tth*PNP I
Ara-A	1.5 mM	260	13,300_Ade_/14,700_araA_	e	*Ec*PNP, *Tth*PNP II

* a. Standard coupled XO procedure; b. Direct spectrophotometric assay; c. HPLC determination: Δt = 8 min, linear gradient CH_3_CN in H_2_O (2–25% for 12 min, 1 mL/min); d. HPLC determination: Δt = 40 min, linear gradient CH_3_CN in H_2_O (1–6% for 12 min, 1 mL/min); e. HPLC determination: Δt = 50 min, linear gradient CH_3_CN in H_2_O (2–12% for 12 min, 1 mL/min). See Supplementary Materials for details.

**Table 2:** Solubility of some natural nucleosides and nucleobases in a neutral aqueous medium [21,23,24].

Compound	Solubility, mM, 25 °C	Solubility, mM, Elevated Temperature (T, °C)	
Adenine	6.6–8.0	13.9 (38)	180.5 (100)
Adenosine	19.2	58.8 (50) *	
Guanine	0.039	0.265 (40)	
Guanosine	1.82	107.3 (100)	
Hypoxanthine	5.29	108 (100)	
Inosine	78.4 **	605 (70) **	

* calculated from [23], ** calculated from [24].

**Table 3:** Comparative characterization of PNPs from different bacterial sources (25 °C for *Ec*PNP and *Ent*PNP, 80 °C for *Tth*PNP I and II, KH_2_PO_4_ buffer 50 mM, pH 7.5).

Enzyme Characteristics	*Ec*PNP	*Ent*PNP	*Tth*PNP	
			I	II
Natural substrates				
Specificity to Ino	+	+	+	--
Specificity to Ado	+	--	--	+
Phosphorolysis of Ino				
Activity (mM/min per mg) *	100% (7.27)	100% (8.37)	100% (67.4)	--
K_M_, µM	76 ± 21 ^1^	107 ± 21	894 ± 196 ^1^	--
k_cat **calc**_, s^−1^ **	29.2	19.1	192.6	--
k_cat_ **_calc_** / K_M_ **	0.38	0.18	0.22	--
Phosphorolysis of Ado				
Activity (mM/min per mg) *	626% (45.5)	--	--	100% (157)
K_M_, µM	21 ± 5 ^1^	--	--	383 ± 132 ^1^
k_cat **calc**_, s^−1^ **	137.7	--	--	482.7
k_cat_ **_calc_**/K_M_ **	6.6	--	--	1.3
Arabinose derivatives				
Specificity to ara-Hx	+	+	+	--
Specificity to ara-A	+	--	--	+
Phosphorolysis of ara-Hx				
Activity (mM/min per mg) *	2.6% (0.186)	0.036% (0.00304)	0.22% (0.150)	--
K_M_, µM	1810 ± 371	2898 ± 831	≥21,500 ***	--
k_cat **calc**_ (k_cat **obs**_), s^−1^ **	0.709 (0.479)	0.0079 (0.0044)	≥2.42 (0.391) ***	--
k_cat_ **_calc_**/K_M_ **	3.9 ×·10^−4^	2.7 ×·10^−6^	~1.1 × 10^−4^ ***	--
Phosphorolysis of ara-A				
Activity (mM/min per mg) *	1.8% (0.815)	--	--	0.057% (0.0902)
K_M_, µM	≥1260 ***	--	--	≥1015 ***
k_cat **calc**_ (k_cat **obs**_), s^−1^ **	≥3.46 (2.10) ***	--	--	≥0.420 (0.271) ***
k_cat_ **_calc_**/K_M_ **	~2.7 ×·10^−3^ ***	--	--	~4.1 ×·10^−4^ ***

-- very poor or not a substrate. * The specific activity is given (mM of substrate/minute per mg of enzyme). The enzyme activity during phosphorolysis of the natural substrate was taken as 100%. ** The minimum value of k_cat_ is given per enzyme molecule, assuming a 100% titer of working active centers. *** The K_M_, k_cat_, and specificity constant values are given as approximate (~, ≥) because the accuracy of their calculation was limited by the range of concentrations tested (kinetic curves without plateauing). ^1^ The kinetic parameters obtained earlier under close experimental conditions can be found in [3,15].

## Data Availability

Data are contained within the article and Supplementary Materials.

## Supplementary Materials

The following supporting information can be downloaded at: https://www.mdpi.com/article/10.3390/biom14091069/s1, Table S1. The influence of organic solvents on the activity of mesophilic and thermophilic PNPs in the reaction of phosphorolysis of inosine (50 mM KH_2_PO_4_, pH 7.5, 25 °C for *Ec*PNP and *Ent*PNP, 80 °C for *Tth*PNP I). Table S2. Parameters for kinetic curves plotting. Enzymatic phosphorolysis (50 mM KH_2_PO_4_, pH 7.5, 25 °C for *Ec*PNP and *Ent*PNP, 80 °C for *Tth*PNP I). Figure S1. Inosine phosphorolysis kinetics. Standard coupled XO procedure. Figure S2. Adenosine phosphorolysis kinetics. Direct spectrophotometric assay. Figure S3. Arabinonucleosides and inosine phosphorolysis kinetics. HPLC assay. Figure S4. ^1^H-NMR spectrum of 9-(β-D-arabinofuranosyl)hypoxanthine in DMSO-*d*_6_. Figure S5. ^13^C-NMR spectrum of 9-(β-D-arabinofuranosyl)hypoxanthine in DMSO-*d*_6_. Figure S6. High-resolution mass spectrum of 9-(β-D-arabinofuranosyl)hypoxanthine. Figure S7. The evaluation of the dynamics of the enzymatic deamination reaction of ara-A. A—starting ara-A; B—reaction, 4 h after ADA adding; C—reaction, 48 h after ADA adding. Figure S8. An example of a chromatogram of the adenosine phosphorolysis catalyzed by PNP *Thermus thermophilus* II. The sample was taken 6 minutes after the initiation of the reaction. Reference [38] is cited in the supplementary materials.
