# Supplementary material for: Bacterial Purine Nucleoside Phosphorylases from Mesophilic and Thermophilic Sources: Characterization of Their Interaction with Natural Nucleosides and Modified Arabinofuranoside Analogues
Source: Biomolecules. 2024 Aug 27;14(9):1069. doi: 10.3390/biom14091069 (PMC11430614; doi:10.3390/biom14091069)
Supplement: Supplementary file 1 [file biomolecules-14-01069-s001.zip › biomolecules-3121144-supplementary.pdf]

SUPPLEMENTARY MATERIALS

Table S1. The influence of organic solvents on the activity of mesophilic and thermophilic PNPs in the reaction of phosphorolysis of inosine (50 mM KH_2PO_4, pH 7.5, 25°C for *Ec*PNP and *Ent*PNP, 80°C for *Tth*PNP I).

**Fraction of organic solvent, v/v, %**		**0**	**5**	**10**	**20**	**30**	**40**
**Solvent**	**Enzyme**	**Relative enzyme activity, %**					
CH_3CN	*Ec*PNP	100	58.1	33.0	13.4	10.5	5.0
	*Ent*PNP	100	96.3	82.7	29.7	2.1	0.0
	*Tth*PNP I	100	70.3	59.1	10.4	0.0	0.0
EtOH	*Ec*PNP	100	123.5	121.1	94.4	43.9	16.8
	*Ent*PNP	100	109.7	104.5	67.0	19.3	2.9
	*Tth*PNP I	100	103.3	92.3	35.6	11.6	2.2
DMF	*Ec*PNP	100	88.8	79.2	60.2	25.9	6.7
	*Ent*PNP	100	92.5	77.4	48.6	23.8	9.7
	*Tth*PNP I	100	90.0	57.4	26.6	7.1	0
DMSO	*Ec*PNP	100	98.2	97.4	86.3	55.4	17.6
	*Ent*PNP	100	89.8	82.7	67.4	46.5	24.3
	*Tth*PNP I	100	90.4	69.5	38.2	10.3	0

Table S2. Parameters for kinetic curves plotting. Enzymatic phosphorolysis (50 mM KH_2PO_4, pH 7.5, 25°C for *Ec*PNP and *Ent*PNP, 80°C for *Tth*PNP I).

Enzyme	Substrate	Method	Number of points	Concentration range, µM
*Ec*PNP, *Ent*PNP	Ino	a	7	50-400
	Ara-Hx	d	9	500-4000
*Ec*PNP	Ado	b	9	20-300
	Ara-A	e	7	250-1500
*Tth*PNP I	Ino	b, c	10	500-15000
	Ara-Hx	d	9	500-4000
*Tth*PNP II	Ado	b, c	9	200-4000
	Ara-A	e	7	250-1500

Initial phosphorolysis rate measurements. Methods.

For the purposes of activity assay and kinetic curves plotting the initial phosphorolysis rate was determined at 25°C for mesophilic and 80°C for thermophilic enzymes in 50 mM KH_2PO_4 buffer, pH 7.5. Concentration of nucleosides in activity assay is presented in Table 1. Concentration of nucleosides in experiments for kinetic curves plotting is presented in Table S2. All reaction mixtures were prepared in the volume of 1 mL and reactions were initiated by addition of PNPs. Observation wavelength is shown in Table 1. The difference in extinction coefficients between the reaction product and the starting nucleoside was obtained experimentally and is shown in Table 1. Measurements for each concentration point were performed at least twice.

Method a. *Standard coupled XO procedure.*

Initial phosphorolysis rate for activity assay and kinetic curves plotting was determined using the coupling assay of Kalckar [38]. The mixtures containing 50 mM KH_2PO_4 (pH 7.5) and 50-400 µM inosine were equilibrated at 25°C and pre-incubated with XO. The reactions were initiated by adding PNP. The increase in the absorption of the reaction mixture due to the formation of uric acid was recorded at a wavelength of 293 nm ($\Delta\varepsilon$ = 12 000). The initial phosphorolysis rates were calculated from the slope of the linear section of the Absorption - Time curves. The kinetic curves were plotted using the mean slope values.

Figure S1. Inosine phosphorolysis kinetics. Standard coupled XO procedure.

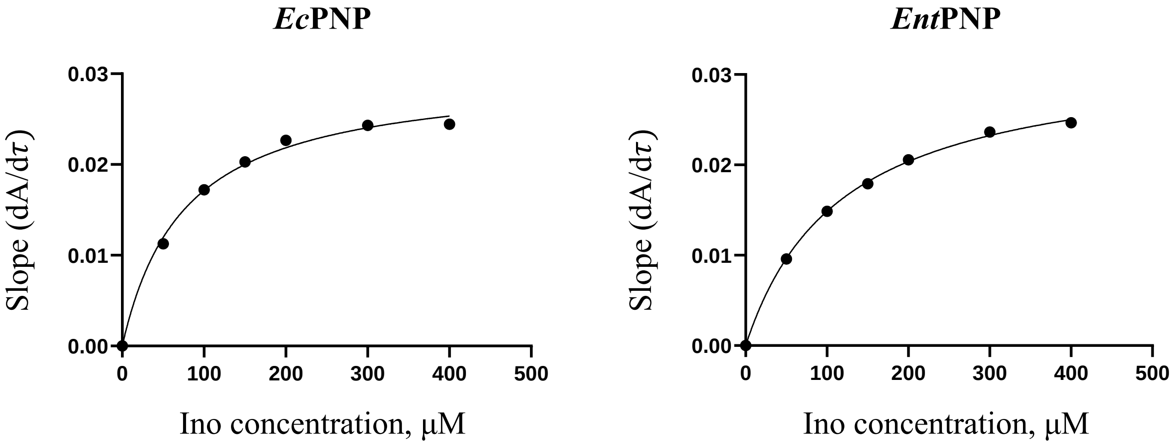

Method b. *Direct spectrophotometric assay.*

Initial phosphorolysis rate for activity assay and kinetic curves plotting was determined by direct spectrophotometric measurement of the decrease in reaction mixtures absorption. The mixtures containing 50 mM KH_2PO_4 (pH 7.5) and adenosine were equilibrated at the appropriate temperature (20-300 µM Ado and 25°C for *Ec*PNP; 200-1000 µM Ado and 80°C for *Tth*PNP II). The reactions were initiated by adding PNP. The decrease in the absorption of the reaction mixture due to the formation of adenine was recorded at a wavelength of 274 nm ($\Delta\varepsilon$ = 1 020). The initial phosphorolysis rates were calculated from the slope of the linear section of the Absorption - Time curves. The kinetic curves were plotted using the mean slope values.

Figure S2. Adenosine phosphorolysis kinetics. Direct spectrophotometric assay.

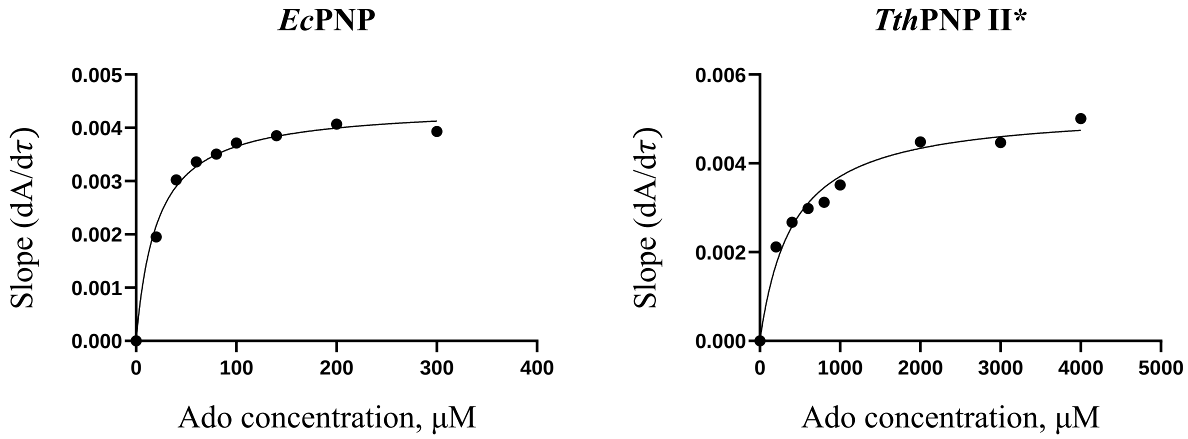

*The figure shows a hybrid graph. The points are obtained by direct spectrophotometric measurement b (200-1000 μM) and by method c (see below, 200-4000 μM).

Methods c-e. *HPLC assay.*

Initial phosphorolysis rate for activity assay and kinetic curves plotting was determined from the change in the peak areas of nucleosides and nucleic bases. For operation within the linear section of the Concentration - Time curve, the degree of phosphorolysis at the moment the reaction stops did not exceed 5%. The kinetic curves were plotted using the mean rate μM/min values.

Method c. The mixtures containing 50 mM KH_2PO_4 (pH 7.5) and nucleoside (500-15000 μM Ino for *Tth*PNP I; 200-4000 μM Ado and for *Tth*PNP II) were equilibrated at 80°C. The reactions were initiated by adding PNP. After incubation at 80°C (8 minutes for Ino and 6 min for Ado), a 200 μl aliquot of the reaction mixture was poured into 600 μl ice-cold CH_2Cl_2, vortexed and centrifuged (13 400 rpm). Samples for HPLC analysis were carefully taken from the upper aqueous layer. The analysis was carried out in a linear gradient of acetonitrile in deionized water from 2 to 25% over 12 min at a flow rate of 1 mL/min and a wavelength of 260 nm.

Method d. The mixtures containing 50 mM KH_2PO_4 (pH 7.5) and hypoxanthine arabinoside, (500-4000 μM) were equilibrated at 25°C / 80°C. The reactions were initiated by adding PNP. After incubation at 25°C / 80°C for 40 minutes, a 200 μl aliquot of the reaction mixture was poured into 600 μl ice-cold CH_2Cl_2, vortexed and centrifuged (13 400 rpm). Samples for HPLC analysis were carefully taken from the upper aqueous layer. The analysis was carried out in a linear gradient of acetonitrile in 0.05% v/v aqueous trifluoroacetic acid from 1 to 6% over 12 min at a flow rate of 1 mL/min and a wavelength of 243 nm.

Method e. The mixtures containing 50 mM KH_2PO_4 (pH 7.5) and adenine arabinoside, (250-1500 μM) were equilibrated at 25°C / 80°C. The reactions were initiated by adding PNP. After incubation at 25°C / 80°C for 50 minutes, a 200 μl aliquot of the reaction mixture was poured into 600 μl ice-cold CH_2Cl_2, vortexed and centrifuged (13 400 rpm). Samples for HPLC analysis were carefully taken from the upper aqueous layer. The analysis was carried out in a linear gradient of acetonitrile in 0.05% v/v aqueous trifluoroacetic acid from 2 to 12% over 12 min at a flow rate of 1 mL/min and a wavelength of 260 nm.

Figure S3. Arabinonucleosides and inosine phosphorolysis kinetics. HPLC assay.

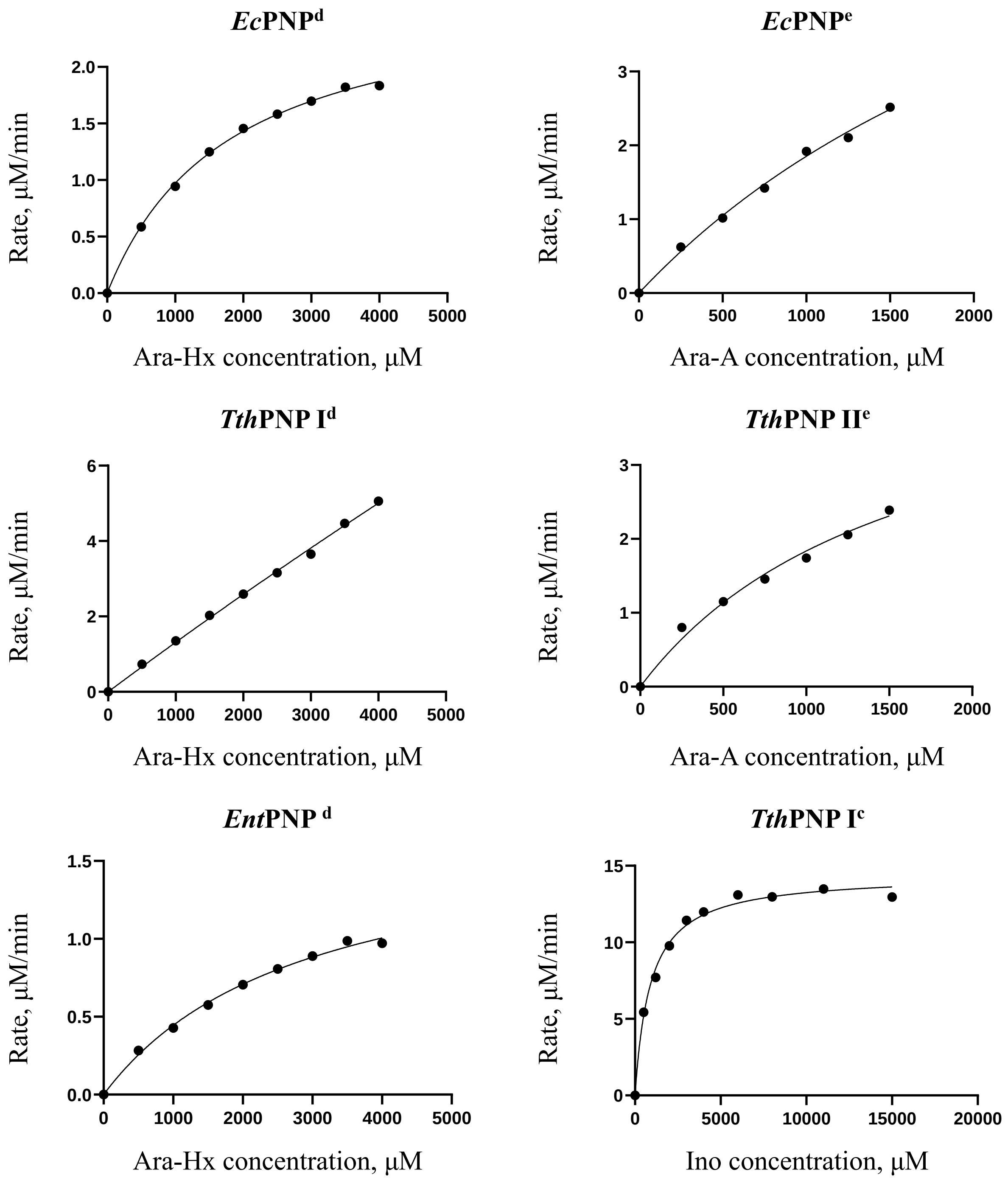

Figure S4. ^{1}H-NMR spectrum of 9-(β-D-arabinofuranosyl)hypoxanthine in DMSO-d_6.

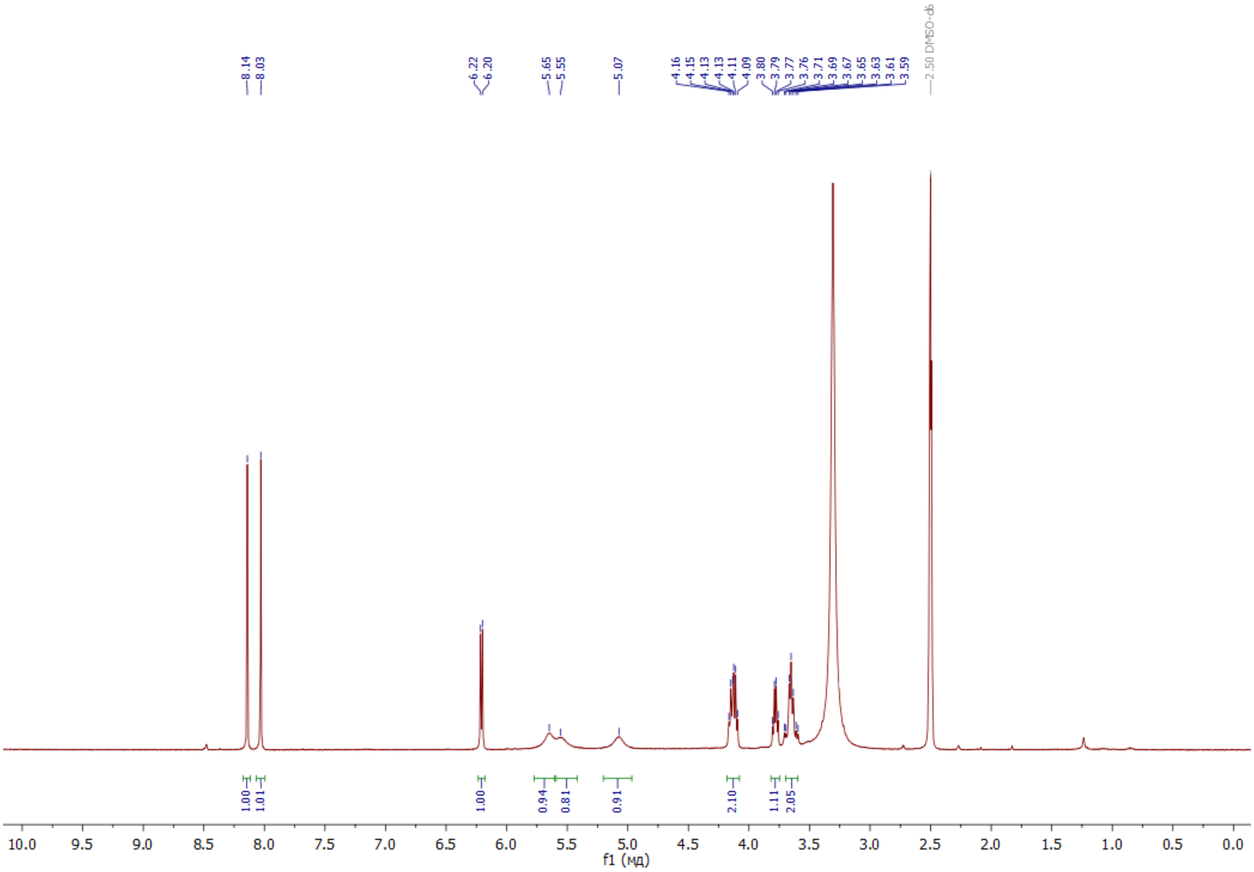

The ^{1}H-NMR spectrum was recorded using a Bruker AVANCE II 300 (Karlsruhe, Germany) instrument at a temperature of 303 K with an operating frequency of 300.1 MHz. The chemical shifts (δ) are given in parts per million (ppm), measured relative to the residual solvent signal as an internal standard (DMSO-d_6, ^{1}H: 2.5 ppm). The spin-spin coupling constants (J) are measured in hertz (Hz). When describing ^{1}H-NMR spectra, the following abbreviations are used: s = singlet, d = doublet, dd = doublet of doublets, m = multiplet.

^{1}H-NMR (300.1 MHz, DMSO-d_6): δ= 8.14 (s,1H, H8), 8.03 (s, 1H, H2), 6.21 (d, 1H, $J_{1',2'}$= 4.8 Hz, H1′), 5.65 (s, 1H, 3′-OH), 5.55 (s, 1H, 2′-OH), 5.07 (s, 1H, 5′-OH), 4.16-4.09 (m, 2H, H3′ + H4′), 3.78 (dd, 1H, $J_{2',3'}$ = 8.9 Hz, H2′), 3.67 (dd, 1H, $J_{5'a,4'}$ = 3.9 Hz, $J_{5'a,5'b}$= -11.7 Hz, H5′ a), 3.63 (dd, 1H, $J_{5'b,4'}$ = 5.0 Hz, H5′ b)

Figure S5. ^{13}C-NMR spectrum of 9-(β-D-arabinofuranosyl)hypoxanthine in DMSO-d_6.

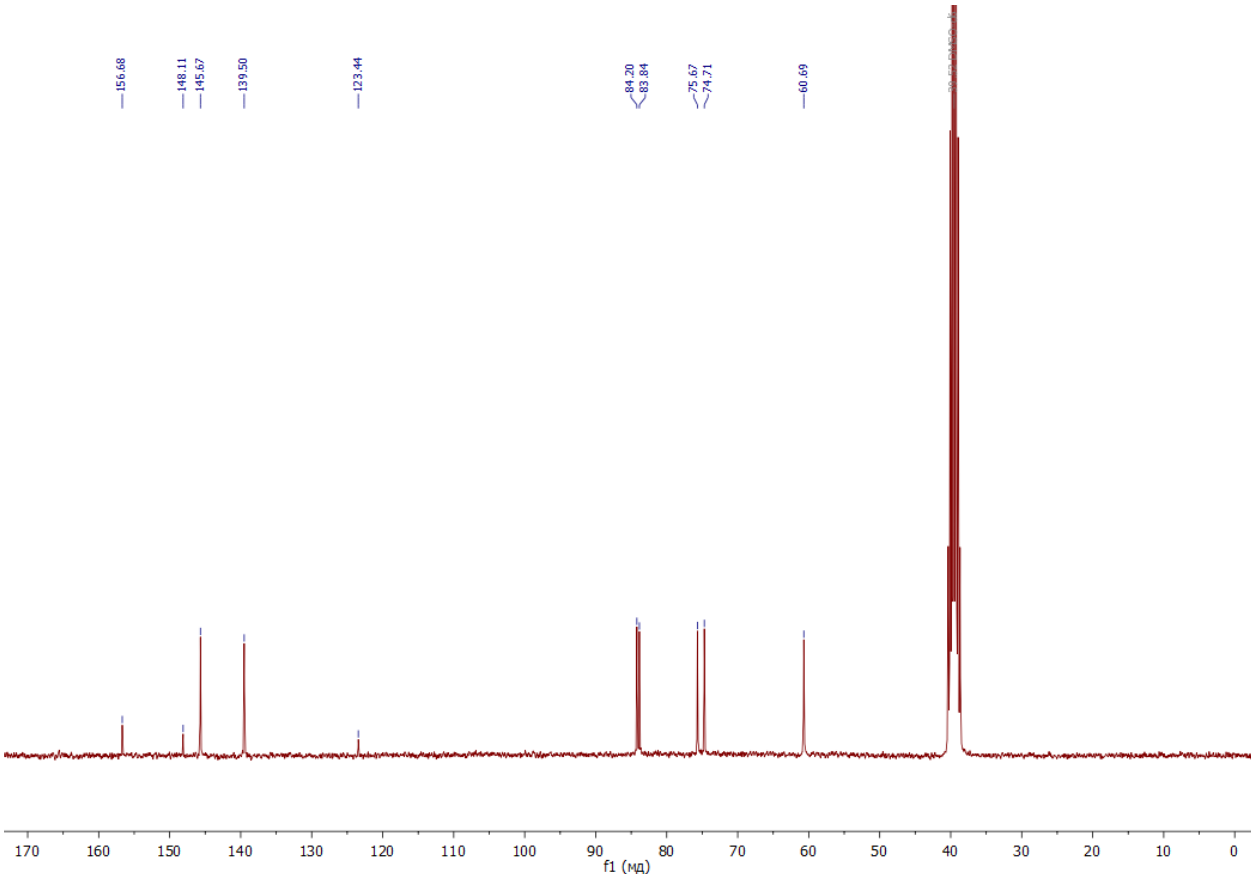

The ^{13}C-NMR spectrum was recorded using a Bruker AVANCE II 300 (Karlsruhe, Germany) instrument at a temperature of 303 K with an operating frequency of 75.5 MHz. The chemical shifts (δ) are given in parts per million (ppm), measured relative to the residual solvent signal as an internal standard (DMSO-d_6, ^{13}C: 39.5 ppm).

^{13}C-NMR (75.5 MHz, DMSO-d_6): δ = 156.68 (C6), 148.11 (C4), 145.67 (C2), 139.50 (C8), 123.44 (C5), 84.20 (C1′), 83.84 (C4′), 75.67 (C3′), 74.71 (C2′), 60.69 (C5′).

Figure S6. High-resolution mass spectrum of 9-(β-D-arabinofuranosyl)hypoxanthine.

Display Report

Analysis Info

Analysis Name	D:\Data\Mikh-298 (ara-I)_5_01_4609.d	Acquisition Date	7/29/2024 7:08:09 PM
Method	la_2.2_small2.m	Operator	BDAL@DE
Sample Name	Mikh-298 (ara-I)	Instrument	compact 8255754.20088
Comment			

Acquisition Parameter

Source Type	ESI	Ion Polarity	Positive	Set Nebulizer	0.4 Bar
Focus	Active	Set Capillary	4500 V	Set Dry Heater	180 C
Scan Begin	50 m/z	Set End Plate Offset	-500 V	Set Dry Gas	4.0 l/min
Scan End	3000 m/z	Set Charging Voltage	2000 V	Set Divert Valve	Source
		Set Corona	0 nA	Set APCI Heater	0 C

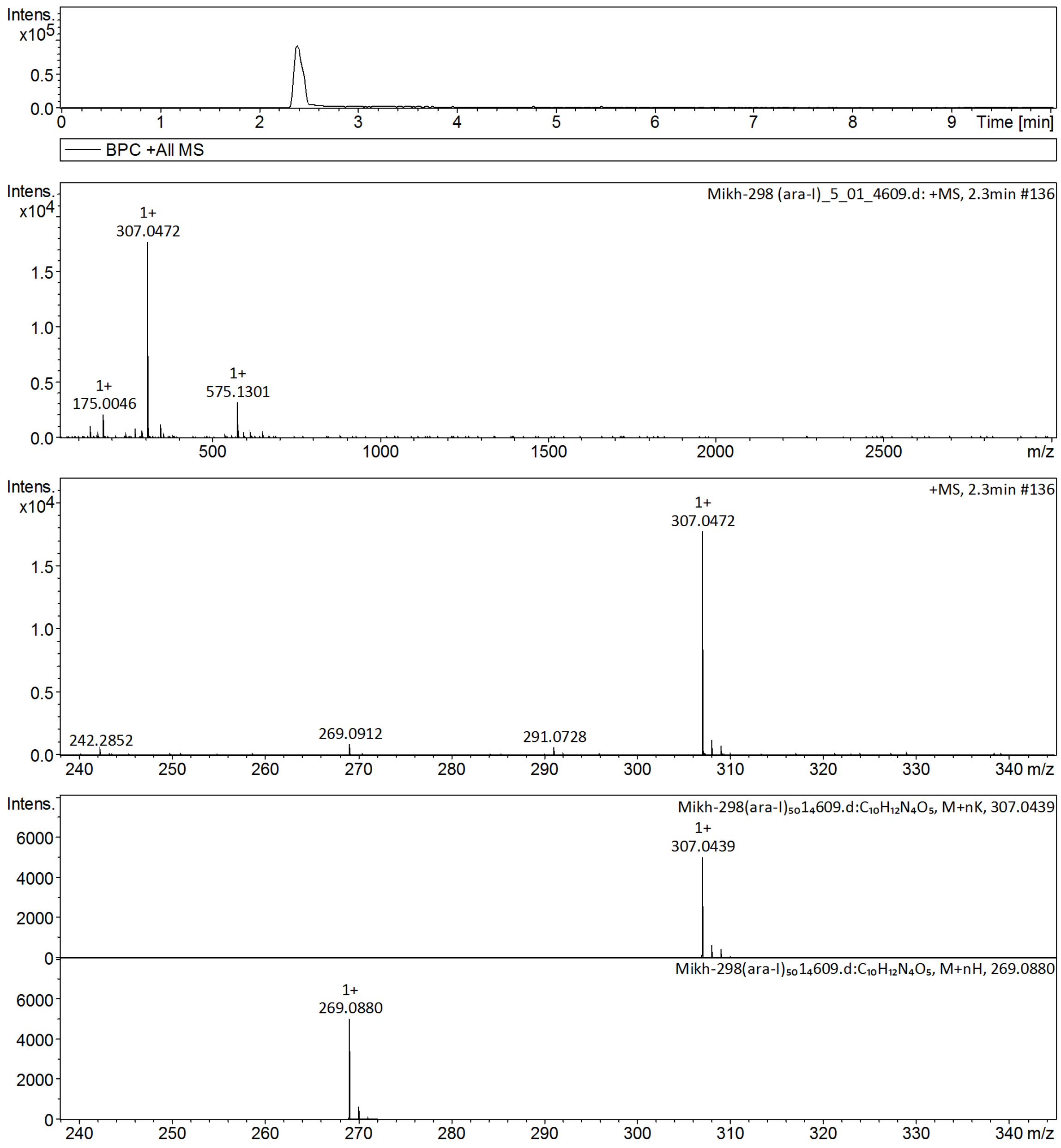

Mikh-298 (ara-I)_5_01_4609.d

Bruker Compass DataAnalysis 4.3 printed: 7/30/2024 2:15:17 PM by: BDAL@DE Page 1 of 1

HRMS: *m/z* $[C_{10}H_{12}N_4O_5+K]^+$ calculated *m/z* 307.0445, found *m/z* 307.0439, $[C_{10}H_{12}N_4O_5+H]^+$ calculated *m/z* 269.0886, found *m/z* 269.0880.

Figure S7. The evaluation of the dynamics of the enzymatic deamination reaction of ara-A. A – starting ara-A; B – reaction, 4 h after ADA adding; C – reaction, 48 h after ADA adding.

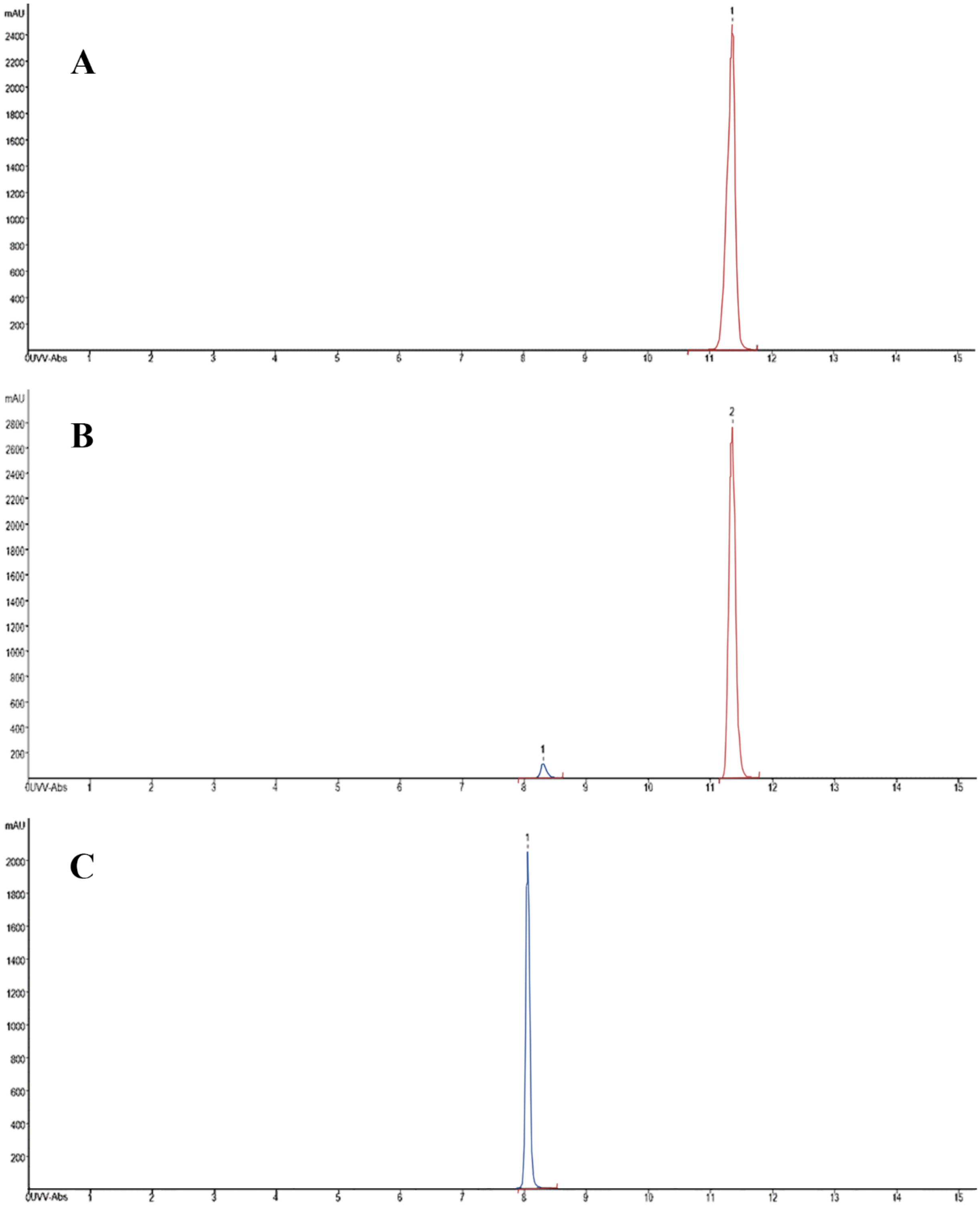

Completion of reaction was assessed by HPLC analysis in a linear gradient of acetonitrile (from 2% to 12%) in deionized water (mQ), a flow rate of 1 mL/min with UV detection at λ 260 nm. Arabinofuranosyl hypoxanthine obtained in a transglycosylation reaction (arabinofuranosyl uracil as an arabinose donor, hypoxanthine as an acceptor) was used as a control.

Figure S8. An example of a chromatogram of the adenosine phosphorolysis catalyzed by PNP *Thermus thermophilus* II. The sample was taken 6 minutes after the initiation of the reaction.

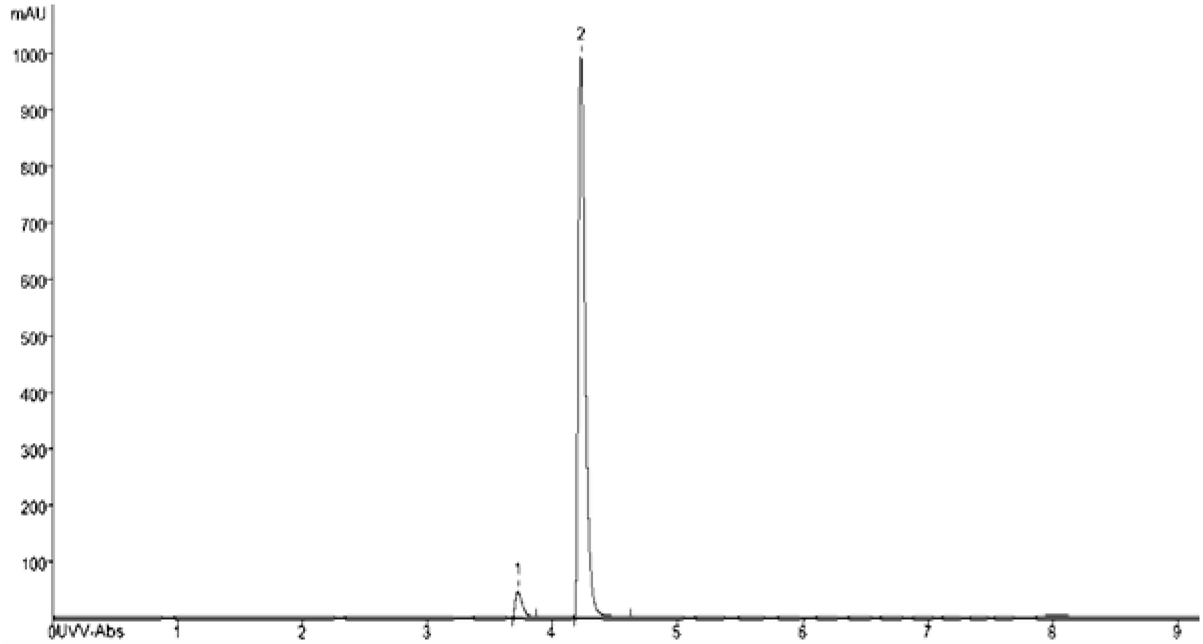

C (Ado)o, µM	400		
Peak (№)	Reaction component	Peak area (S), mV*s	RT, min
1	Ade	171.6	3.7
2	Ado	3863.0	4.2

Reagents and conditions: 400 µM Ado, *Tth*PNP II, 80°C, 50 mM KH_2PO_4 buffer, pH 7.5. Enzymatic reactions were assessed by HPLC analysis in a linear gradient of acetonitrile (from 5% to 30%) in deionized water (mQ), a flow rate of 1 mL/min with UV detection at λ 260 nm.
